# Grammatical Evolution of Complex Digital Circuits in SystemVerilog

**DOI:** 10.1007/s42979-022-01045-9

**Published:** 2022-03-12

**Authors:** Michael Tetteh, Douglas Mota Dias, Conor Ryan

**Affiliations:** 1 University of Limerick, Limerick, Ireland; 2 Rio de Janeiro State University, Rio de Janeiro, Brazil

**Keywords:** Grammatical evolution, Evolvable hardware, Evolutionary design of conventional circuits, Hardware description language (HDL), Verilog, SystemVerilog, Register transfer level (RTL), Combinational circuits, Sequential circuits, Corner case testing

## Abstract

The evolution of complex circuits remains a challenge for the Evolvable Hardware field in spite much effort. There are two major issues: the amount of testing required and the low evolvability of representation structures to handle complex circuitry, at least partially due to the destructive effects of genetic operators. A 64-bit $\times$ 64-bit add-shift multiplier circuit modelled at register-transfer level in SystemVerilog would require approximately 33,200 gates when synthesized using Yosys Open SYnthesis Suite tool. This enormous gate count makes evolving such a circuit at the gate-level difficult. We use Grammatical Evolution (GE) and SystemVerilog, a hardware description language (HDL), to evolve fully functional parameterized Adder, Multiplier, Selective Parity and Up–Down Counter circuits at a more abstract level other than gate level—register transfer level. Parameterized modules have the additional benefit of not requiring a re-run of evolutionary experiments if multiple instances with different input sizes are required. For example, a 64-bit $\times$ 64-bit and 128-bit $\times$ 128-bit multipliers etc., can be instantiated from  a fully evolved functional and parameterized N-bit $\times$ N-bit multiplier. The Adder (6.4$\times$), Multiplier (10.7$\times$) and Selective Parity (6.7$\times$) circuits are substantially larger than the current state of the art for evolutionary approaches. We are able to scale so dramatically because of the use of a HDL, which permits us to operate at a register-transfer level. Furthermore, we adopt a well known technique for reducing testing from digital circuit design known as corner case testing. Skilled circuit designers rely on this to avoid time-consuming exhaustive testing. We demonstrate a simple way to identify and use corner cases for evolutionary testing and show that it enables the generation of massively complex circuits. All circuits were successfully evolved without resorting to the use of any standard decomposition methods, due to our ability to use programming constructs and operators available in SystemVerilog.

## Introduction

Researchers began to explore the feasibility of the application of evolutionary algorithms (EAs) to circuit design tasks back in the early 1990s [2–4], which gave birth to the evolvable hardware (EHW) field. The choice of EAs for circuit evolution is motivated by the fact that EAs were demonstrated to be better and more creative at exploring the circuit design search space than humans [5].

EHW is made up two main sub domains: adaptive hardware and evolutionary design of conventional digital circuits [6]. The latter, adaptive hardware, refers to the continuous and autonomous reconfiguration or adaptation of evolved hardware to conform to changing operational requirements or conditions of its deployed environment over its lifespan [6, 7]. On the other hand, the latter deals with the design of conventional digital circuits. EAs such as Genetic Algorithm, Genetic Programming, Cartesian Genetic Programming (CGP), Grammatical Evolution (GE) etc. have been applied to circuit design tasks.

The ultimate objective of EHW is the development of next generation hardware that is capable of self-reconfiguration through evolution [6, 8]. However, a major and basic requirement of an adaptive hardware system is the ability to evolve its circuitry. A major reason for the extreme importance of adaptive systems is their suitability for use in environments characterized by extreme conditions (such as extreme temperatures, high radiations etc.), which are unsafe and inaccessible to humans to operate. A typical application area is space exploration [9–11].

In spite of the immense progress made in the EHW field since its inception, issues of scalability have long hampered its progress. This is because existing methods mostly design circuits at the gate-level using EAs. A major benefit of gate-level evolution is its capability to evolve optimized novel circuit designs (i.e. circuits using fewer gates) compared to human designed circuits. For example, a 3-bit $\times$ 3-bit and a 4-bit $\times$ 4-bit multiplier evolved in [12] were 23.3% and 10.9% more efficient in terms of the number of two input logic gates used respectively. However, the drawbacks of gate-level evolution are: inability to scale to evolve complex circuits [13], designs are less interpretable, difficult and take more time to verify etc. These drawbacks account for the two major issues confronting EHW.

The two major issues are: Scalability of Representation and Scalability of Fitness Evaluation [14]. As circuit inputs increases, longer chromosomes are required to represent circuits. This results in large search spaces which are difficult, slow and expensive to search [14], reducing the chances of EAs finding novel circuit designs. Furthermore, due to the destructive nature of genetic operators, evolving circuits of such complexity becomes intractable. This is termed as Scalability of Representation. Also, increasing circuit inputs increases the number of test cases [15]. As a result, exhaustive testing is only feasible for modestly complex circuits —circuits with a few number of inputs and reasonable number of gates that can be exhaustively tested. This phenomenon is termed Scalability of Fitness Evaluation.

Conventionally, hardware engineers design circuits either through the use of schematic diagrams or Hardware Description Languages (HDLs). Schematic diagrams involve the use of graphical representations of circuit components to design circuits. Schematic diagrams reveal the functionality and connections between the various components used to realize the circuit functionality. HDLs are programming languages used for the behavioural description of circuits. The two dominant HDLs in industry today are Verilog/SystemVerilog (SV) and Very High-Speed Integrated Circuit Hardware Description Language (VHDL).

HDLs are well suited and ideal for large and complex circuits designs compared to schematic diagrams [16, 17]. The use of HDLs is the *de facto* standard in the design of digital circuits in industry due to a number of reasons: first, real world digital circuit designs are usually intractable to design at the gate-level; second, more abstract designs (such as RTL) are more interpretable, require relatively less time to vary/adapt and easier to verify [20]; third, they enable design module reuse through parameterization and support for mixed-style design in a single module; fourth, HDL designs are portable across different hardware because they can be synthesized for several vendor-specific Field Programmable Gate Arrays (FPGAs) [16] , unlike other approaches which evolve circuits directly on a specific FPGA, such as Xilinx XC6200 series, by evolving bitstreams that configure the FPGA [21]. These approaches stalled after Xilinx Corporation discontinued the production of such FPGAs due to cost and security reasons [21].

HDLs support digital circuit designs at different levels of abstraction. For example, in SystemVerilog, circuits can be designed as low as switch-level, gate-level, register-transfer level (RTL) and as high as behavioural level. To date, the most complex fully functional circuits evolved are: a 10-bit + 10-bit adder [22], a 6-bit $\times$ 6-bit multiplier [15], a 19-bit parity circuit [23] and a 28-input frg1 circuit [24].

In this paper we seek to answer the following research questions:

1. Can the use of HDLs (SystemVerilog in this case) evolve complex combinational and sequential circuits that compare favourably to state-of-the-art methods in terms of the size of inputs and gate count?
2. What are the drawbacks of evolving circuits using GE and HDLs?

This paper is an extension of the work published under the title: “Evolution of Complex Combinational Logic Circuits Using Grammatical Evolution with SystemVerilog” in European Conference on Genetic Programming 2021 [25]. The initial paper examined the concept of evolving complex and parameterized combinational circuits in SystemVerilog using GE with behavioural constructs. Additionally, corner case testing was employed to significantly reduce the amount of testing, since exhaustive testing is not feasible for complex circuits. This paper builds upon this work by

- Evolving a parameterized sequential circuit—Mod-N Up-Down Counter;
- Synthesizing and comparing of evolved parameterized circuits to the state-of-the-art works;
- Investigating the effect of varying population sizes on evolutionary performance;
- Performing statistical analysis on the effect of input bit-width sizes on simulation time, to determine appropriate input bit-width sizes for evolving parameterized circuits.

In summary, this paper tackles the issues of scalability through the use of SystemVerilog with GE to design fully functional and complex combinational circuits. This is achieved without the use of decomposition. The main contributions of this paper are:

1. Evolution of fully complex functional combinational digital circuits: 64 $\times$ 64-bit multiplier, 64 + 64-bit adder, 128-bit selective parity circuit and 64-bit Up–Down Counter;
2. All evolved circuits are parameterized. Thus, these design modules are reusable and only require the specification of input and output bit-width sizes without the need for re-run of experiments. The bit-width sizes mentioned in (1) above are the default bit-width sizes for the evolved circuits;
3. A significant reduction in the amount of testing through appropriate choice of operators and the combination of corner case and randomized testing in testbenches. Specifically, $3.40 \times 10^{36}$, $6.80 \times 10^{36}$, $2(6.80\times 10^{36})$ and $2.09 \times 10^{38}$ test cases reduction for Adder, Multiplier, Selective Parity and Mod-N Up–Down Counter circuits respectively.

## Background

There are two main types of digital circuits, namely, combinational and sequential. Combinational circuits are a class of circuits whose output(s) depends solely on current inputs. Sequential circuits on the other hand, are a class of circuits whose output(s) depends on both current and past input(s). Sequential circuits consist of memory elements such as flip-flops which store the output of combinational logic which are then fed back as input. The vast majority of circuits evolved in EHW literature are combinational. This is partly due to complex circuit connections and unavailability of appropriate encoding structures for chromosomes that model feedback loops of sequential circuits [13]. For example, in [26], CGP was modified to allow levels forward in order to model feedback loops of sequential circuits.

Cartesian Genetic Programming (CGP) is a GP variant widely used in EHW [27]. CGP uses direct acyclic graphs to represent its phenotype [28]. A CGP genotype consist of function, connection and output genes which encode a node’s operation, its source of input(s) and the program output locations respectively. CGP is a well known technique for the evolution of digital circuits and much interesting progress has been made since its inception [29]. CGP has not only been used to evolve a range of fully functional circuits at the gate-level, but also at the functional-level. It has also been applied to the design of approximate circuits [30]. Approximate circuits is a category of circuits which trades off hundred percent functionality for other equally important circuit parameters such as area, power consumption etc.

### Grammatical Evolution

GE is a grammar-based GP variant which evolves programs in any Backus-Naur Form compliant language [31]. GE uses a mapper which employs a linear genome consisting of integer codons and a grammar describing a subset of the target language to generate programs. A modulo rule is used to dictate production rule selection. The process expands all non-terminals to terminal symbols. A valid program or phenotype is obtained once all non-terminals have been expanded to terminals. GE has been applied to several domain problems such as circuit design [32], symbolic regression [33], Explainable AI [34], Software Testing [35] etc.

Despite its success in many application domains, GE hasn’t been applied often to circuit synthesis problems, although the availability of HDLs makes it an ideal tool for circuit design tasks. An initial exploration of this idea evolved a 1-bit full adder using GE in [36]. Other simple circuits have also been evolved at the gate level [37]. Both studies did so using a HDL. In [32], GE was used to design circuits such as 11-bit Multiplexer, Seven Segment Display and Hamming Code (7,4) Decoder using SystemVerilog. The work also explored the effect of grammar design with and without the incorporation of simple domain knowledge to guide the evolutionary search towards regions of the solution space where optimal solutions are perceived to be.

### SystemVerilog

SystemVerilog is one of the major HDLs used in industry for the design of digital circuits. SystemVerilog is an extension of Verilog—essentially, a superset of Verilog. The extension was based on two objectives: efficient and more accurate modeling of digital circuits functionality; writing of efficient and race free verification code for large and complex designs [20]. Digital circuit design in SystemVerilog can be performed at four main abstraction levels, in decreasing order of abstraction: behavioural level, register-transfer level (RTL), gate level and digital switch level [20]. The higher the abstraction level, the less detail it reveals about the actual circuit representation.

Digital switch level deals with the design of circuits at the transistor level, while gate-level modelling involves the use of only primitive logic gates such as: and, or, nor, xor, not etc. to design circuits. RTL designs make use of two primary constructs: continuous assignments and always procedural blocks [20]. A statement preceded by the assign keyword is a continuous assignment. For example, assign out = inA & inB;. The output signal (out) is continuously driven by the right hand expression whenever any of the operands or signals change, triggering a re-evaluation of the right hand expression. RTL designs obscure circuit functionality realization in silicon which are only revealed after synthesis [20]. In comparison to gate-level designs, RTL designs are interpretable, more capable of dealing with huge input sizes, better suited for verification of large and complex designs [20] etc. Behavioural level models circuits at a higher abstraction level than RTL, utilizing all constructs in SystemVerilog and therefore may not be synthesizable.

#### SystemVerilog Instructions

An always procedural block can be used to model combinational and sequential logic [38]. An always block behaves like an infinite loop that continuously executes statements within its block, except it is triggered either by a time or event control. With time control, the always block repeatedly executes each time the specified delay time elapses. For example, an always block that toggles a clock (clk) signal after every ten time units will be described in SystemVerilog as $\mathtt{always \#10 clk = \sim clk;}$. Note, the time delay syntax is $\mathtt{\#\langle time-unit\rangle }$. An always block with event control requires a *sensitivity list*. A sensitivity list is a list of signals that trigger the execution of the always block whenever any of the signal changes. For example, $\mathtt{always @(signalA, signalB) begin \langle stmts\rangle end}$ and $\mathtt{always @(posedge clk) begin \langle stmts\rangle end}$ for combinational and sequential logic modelling respectively. The syntax for sensitivity list specification is: $\mathtt{@(\langle sensitivity-list\rangle )}$. SystemVerilog supports other specialization of the always block for specific use cases [38].

SystemVerilog has various categories of operators: bitwise, reduction, conditional, arithmetic, logical operators etc. Another very useful SystemVerilog programming construct which we make use of in this work is generate for-loop (synthesizable for-loop), which is useful for the description of circuits with a fixed and repetitive structure. The generate for-loop instruction creates *n* module instances which can be specified through SystemVerilog parameters. SystemVerilog also has switch-case and if-else constructs, which are either synthesized to multiplexers or priority encoders by synthesis compilers based on mutual exclusivity of the selection items [38]. However, there exist modifiers to use in conjunction with switch-case and if-else statements to reduce ambiguities and ease the task of synthesis compilers [39].

## Related Work

Research conducted to address scalability issues in evolutionary design of digital circuits has followed three main trajectories: Functional-Level Evolution, Increasing evolvability through the improvement of genetic operators and Problem decomposition.

### Functional-Level Evolution

Functional-Level Evolution (FLE) uses secondary logic functions such as adders, multiplexers, multipliers etc. (usually in conjunction with primitive logic gates) other than only primitive logic gates in designing digital circuits [3].

In [40], half-adder, full adder and multiplier circuits were used as functions to design adders and multipliers. In comparison to gate-level evolution, function-level evolution obtained higher success rate as well as significant reduction in the number of generations in some instances [40]. Three-bit multipliers were evolved using binary multipexers in [41] and a proposed Constrained CGP evolved higher order approximate multipliers using imprecise lower order multipliers [42]. Approximate 9-input and 25-input median circuits [43], and an image filter [44] have also been evolved at functional-level.

### Increasing Evolvability Through The Improvement of Genetic Operators

Evolutionary performance is largely dependent on the evolvability of genetic operators. Thus the ability of genetic operators to create offsprings with better or improved fitnesses than their parents. The traditional CGP setup uses point mutation as its variation operator. This incurs wasted evaluations and stalls evolution when point mutation operations affect only inactive genes. Single Active Mutation (SAM), unlike point mutation, ensures an active gene is mutated whenever mutation takes place [45].

Biased SAM is an improvement upon SAM, which increases its robustness when tackling combinational circuit designs. Biased SAM requires a transition probability matrix to work with. This is constructed for each problem by conducting preliminary experiments on the problem. During these initial experiments, functional mutations that increase the overall fitness of an individual are recorded. Subsequently, during the actual experiments, roulette wheel selection is applied on the transition probability matrix to determine the function the chosen functional node should be mutated to [45]. On selected circuit design benchmarks, Biased SAM outperformed SAM.

Guided Active Mutation (GAM), another CGP mutation operator was designed to mutate active node (s) in a subgraph corresponding to a single output [46]. The objective was to increase the overall fitness score of this output when compared to the truth table of the circuit. However, though GAM recorded fewer fitness evaluations to find feasible solutions, it recorded low success rates. As a result, SAM and GAM were merged in order to harness the strengths of both mutation operators. Their combination resulted in high success rates and best results on the selected benchmark problems [46].

Semantically-oriented mutation operator (SOMO) performs mutation in the phenotypic space after which the resultant phenotype is encoded to its corresponding genotype [22]. SOMO randomly selects an active node for mutation. A mutation operation may affect a node function or a node input connection. During node input connection mutation, the best connection point that results in an increase of individual’s fitness is determined through the application of semantics [22]. SOMO evolved combinational circuits significantly faster compared to the multi-threaded parallel CGP implementation in [47].

### Circuit Decomposition Methodologies

Several methodologies have been proposed to decompose complex circuit problems into subcircuits of evolvable complexity. These fall into two main categories: inputs and outputs decomposition. The former refers to decomposition methods that breakdown circuits into subcircuits using only the circuit’s inputs while the latter uses the circuit’s outputs.

Increased complexity evolution (ICE), also referred to as divide and conquer methodology, breaks a complex system into sub-systems of evolvable complexity before evolution is performed on each sub-system in a bottom–up manner [48, 49]. Sub-systems can be evolved in parallel (if no dependency exists between them) or sequentially. These evolved sub-systems serve as building blocks in a more complex sub-system(s) in an upper layer. One challenge with ICE is that it requires the fitness functions for the respective sub-systems to be manually defined. However, for certain problems such as circuit design, a solution to this challenge is either the partitioning of training vectors based on circuit outputs or the training set can be partitioned [48, 49].

In contrast to ICE, Bidirectional Incremental Evolution (BIE) performs automatic decomposition of a complex circuit into subsystems using either a suitable standard decomposition technique (such as Shannon’s theorem) or an EHW-oriented decomposition technique; or a combination of both [50]. BIE progressively decomposes complex circuits into subcircuits in a top-down manner while evolving each subsystem. Subsystem(s) undergo further decomposition only if evolution is unable to obtain optimal solutions. During the second phase of BIE, evolved subsystems are merged and optimized in a bottom–up fashion. In comparison to direction evolution, BIE performed best on 7-inputs and 10-output circuit benchmark problems.

On more complex circuits, BIE performed poorly [15]. As a result, instead of output decomposition in the case of BIE, an input decomposition technique termed Generalized Disjunction Decomposition (GDD) was proposed in [15]. GDD automatically decomposes a complex circuit into two subcircuits: the evolvable and multiplexer subcircuits [15]. GDD requires the user to specify the number of inputs for the evolvable circuit, subject to the constraint that it must be less than the number of inputs of the original circuit. GDD then applies an algorithm to generate the evolvable subcircuit’s outputs using the actual circuit’s truth table. The evolvable subcircuit can be evolved using any EHW methods (such IEC or BIE) without any restrictions. The multiplexer subcircuit takes the outputs of the evolvable subcircuit as inputs and uses the remaining inputs of the actual circuit not used in the evolvable circuit as select lines for the multiplexer. The output of the multiplexer circuit is the same as that of the complete (original) circuit. Using a selection of circuit benchmark problems from Microelectronics Center of North Carolina (MCNC) benchmark suite and randomly generated circuit problems, compared to BIE, GDD required fewer number of generations, time and better fitness scores [15].

Stepwise Dimension Reduction (SDR) is a layered and output decomposition methodology that decomposes a circuit into two subcircuits. One of these is evolved to handle input combinations with an output value of 1 while the second is evolved to handle those with 0 as output [23]. Internal intermediate mappings are devised if subcircuits are not of evolvable complexity. SDR evolved a 19-bit circuit which GDD was unable to evolve. SDR obtained comparable results for few of the benchmark problems in less time compared to GDD, but not on more complex multiple output circuit benchmarks [23]. SDR is effective on single output circuit problems such as the parity circuit.

Common to all these approaches is that they are capable of evolving relatively complex circuits as it is feasible to first evolve subcircuits before merging them into a complete circuit with a high input dimension.

### Other Scalability Approaches

In other works such as [47], multi-threaded parallel implementations of evolutionary algorithms, specifically CGP, have been developed to exploit modern processor architectures to allow for more fitness evaluations. However, just a single run was performed to evolve 5-bit $\times$ 5-bit multiplier due to the associated high computational effort [47]. In  [24], CGP candidate circuits are transformed into a Binary Decision Diagram (BDD). Similarly, the circuit specification is also specified in BDD format. A BDD evaluation is performed, where functional similarity is performed between the candidate circuit’s BDD representation and circuit’s specification also in BDD format. The fitness of the candidate circuit is the hamming distance between the output vectors of the BDD representations of the candidate circuit and circuit specification. Results reveal that BDD-based evaluation is faster than exhaustive testing [24] (Table 1).

**Table 1:**
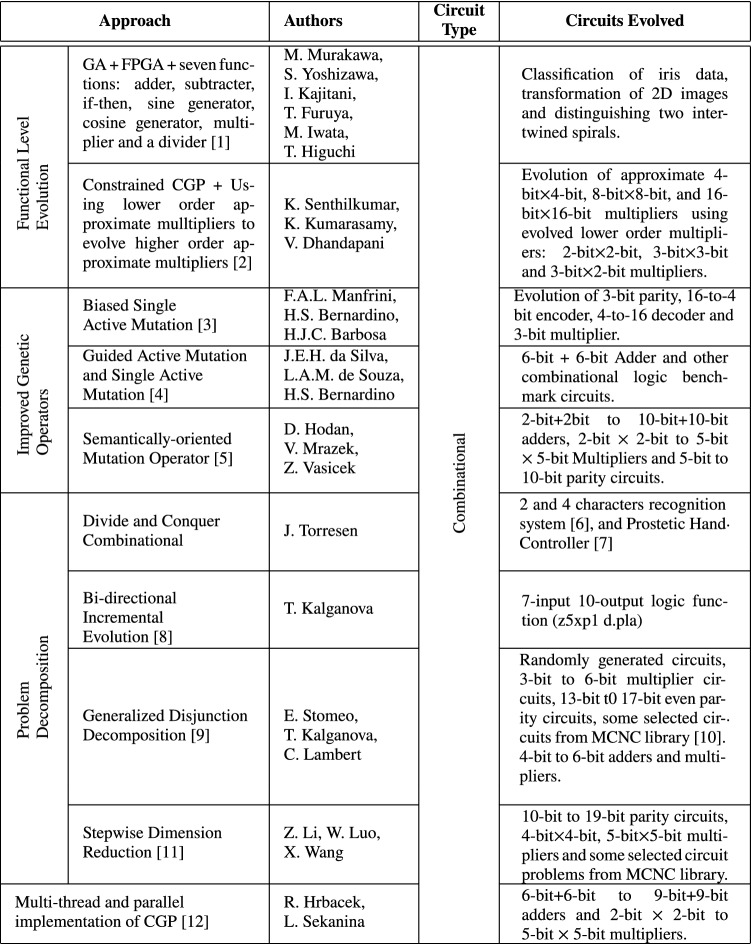
Summary of some proposed approaches to address scalability challenges in EHW

## Experimental Design

We evolve four complex circuits: three combinational and one sequential circuit. The combinational circuits are: multiplier, adder and selective parity generator. The chosen sequential circuit is Up–Down Counter. These problems are representative of evolutionary circuit design benchmark problems in current literature [22, 23, 26, 47]. All circuits are evolved as parameterized design modules to accommodate all possible range of values of the parameters. Thus, evolved parameterized circuit modules retain their functionality for all possible range of values of the specific parameter(s). For example, an evolved optimal parameterized adder (N-bit + N-bit adder) must function perfectly as 8-bit + 8-bit adder, 32-bit + 32-bit adder etc. Prior to simulation or synthesis, parameter values of evolved parameterized circuits can be specified without requiring a re-run of evolutionary experiments, as is the case for existing EHW methodologies.

Parameterized circuits are designed using the keyword parameter in SystemVerilog. The usage of the parameter keyword is highlighted in the corresponding production of $\langle design-module\rangle$ rule for all benchmark circuit grammars. The grammars are divided into two segments separated by a dashed line. The top segment consist of rules that require expansion. In other words, the evolvable part of the grammar. The bottom segment (fixed grammar par) consist of rules that describe the corresponding circuit’s interface, variable/register declaration and initialization, required programming constructs (e.g. always block, generate for-loop) etc. The bottom segment can be left out of the grammar as all the rules consist of single productions. Alternatively, a circuit module template can be designed with a placeholder to represent the evolved segment.

**Fig. 1:**
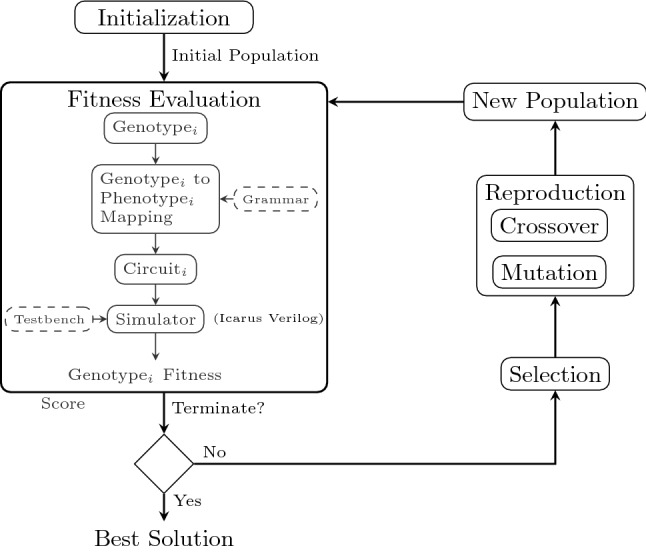
GE functional simulation circuit design process

The GE circuit design process illustrated in Figure 1 is summarized as follows:

1. A randomly generated initial population is created.
2. For each individual in the population, GE mapper uses the specified subset grammar defined for the problem to derive the HDL code (phenotype). The phenotype (resultant circuit) is tested using the simulator (Icarus Verilog). The simulator uses the testbench to test the functionality of the evolved circuits. The total number of training cases passed by an individual represents its fitness score.
3. The termination criterion is checked to determine whether to terminate the experimental run. A termination criterion can be the maximum number of generations or fitness evaluations. If the termination criterion is met, the experimental run is terminated and the individual with the highest aggregated fitness score is returned as the best solution.
4. However, if the termination criterion has not been satisfied, individuals undergo a selection process. Usually, individuals with high fitness scores are chosen for reproduction.
5. The selected individuals undergo crossover and mutation to create offsprings that form a new population.
6. The new population of individuals undergo fitness evaluation (back to step 2).
7. The evolutionary cycle continues until the termination criterion is met and the best candidate solution returned.

### Benchmark Problems

#### Selective Parity Circuit

A parity generator circuit generates a single bit (either 0 or 1) from a string of bits in order to obtain an even or odd number of 1s. An odd parity circuit generates a bit value of 1 and 0 when the data to be transmitted contains an even and odd number of 1s respectively. Conversely, an even parity circuit generates a 0 and 1 when the data to transmitted contains an even and odd number of 1s.

We design two different parity generator circuit grammars: Parity Grammar A and Parity Grammar B, shown in Listings  1 and  2 respectively. The purpose of designing two separate grammars using different operators is to investigate the benefit of using tuned operators in grammars. Selective Parity Grammar A uses generate loops (synthesizable $\langle for-loop\rangle$) and bitwise operators ($\langle bitwise-op\rangle$) to loop through the data bits while performing bitwise operations until a parity bit is obtained. Selective Parity Grammar B uses both reduction operators ($\langle reduction-op\rangle$) and bitwise-operators ($\langle bitwise-op\rangle$). Reduction operators apply bitwise operations on the bits of an $n-bit$ operand (a vector) recursively to produce a scalar output.

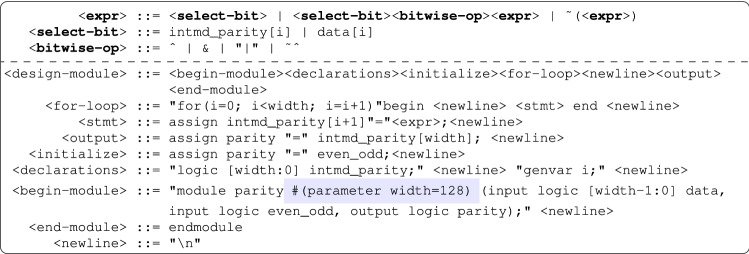

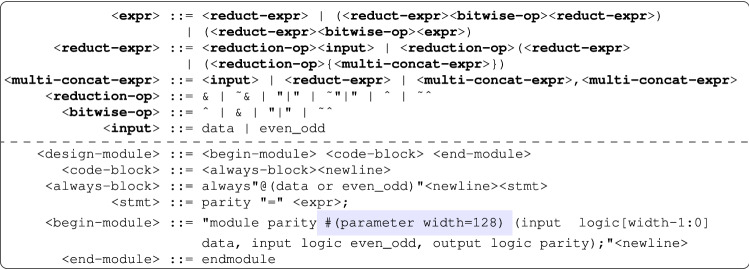





#### N-bit $+$ N-bit Adder

An adder circuit performs addition in digital electronic devices. The adder grammar is shown in Listing 3 and uses the always ($\langle always-block\rangle$) procedural block, which takes a sensitivity list as arguments and executes the statements within its code-block whenever a signal within the list changes. The operators used are binary arithmetic operator ($+$) and bitwise operators (${ \& }, \, {|}, \, {^\wedge }$). The default input bit-width of the addends is 128 specified using the parameter keyword in the $\langle begin-module\rangle$ rule.

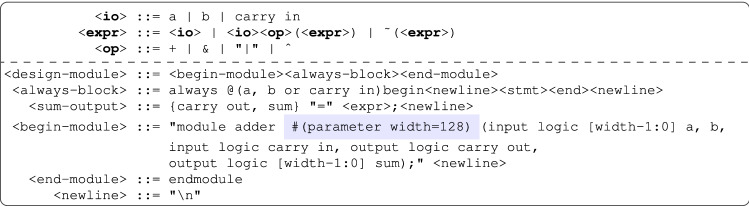

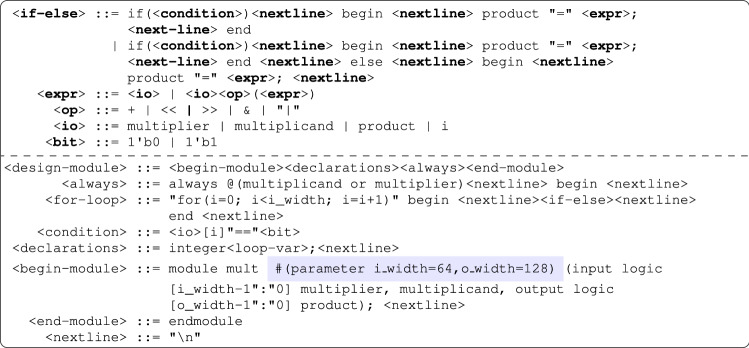





#### N-bit $\times$ N-bit Multiplier

A multiplier performs multiplication in digital electronic devices such as computers, calculators, etc. The particular multiplier type considered here is the Add-Shift Multiplier. The Add-Shift Multiplier’s operation is based on longhand multiplication. Each digit of the multiplier multiplies the multiplicand to obtain an intermediate product shifted a digit to the left of the preceding intermediate product. All intermediate products are then summed up to obtain the product of the multiplication. The Multiplier Grammar is shown in Listing 4. The grammar makes use of always ($\langle always-block\rangle$), for-loop ($\langle for-loop\rangle$) and if-else ($\langle if-else\rangle$) programming constructs in SystemVerilog. Three different operators ($\langle op\rangle$) are used: *binary arithmetic operator *($+$), shift operators (${<<}, \, {>>}$) and bitwise operators (${ \& }, \, {|}$).

### N-bit Up–Down Counter

Counters are sequential logic devices that store the number of occurrences of an event, usually from a pre-defined state. The counting operation is triggered by a clock signal. A counter that increases its content after every clock cycle is known as an up-counter, while one that decreases its content is referred to as down-counter. A bi-directional counter functions as an up and down counter.

An N-bit Up–Down Counter is a counter that functions as a mod-$2^n$ Up-Down Counter. That is, it can function as a mod- 2, 4, 8, 16, 32 etc. Up–Down Counters. The transition table for N-bit Up-Down Counter is captured in Table 2. The counter has an active low-reset signal; thus it resets to State 0 when the reset signal is 0. The Enable_Load input triggers a specified input value to be loaded by the counter. The Enable_Up and Up–Down inputs control the type of counting operation to be performed by the counter. If both inputs are high, the counter behaves as an up-counter. If Up–Down input is high or 1 and the Enable_Up input is low or 0, the counter behaves as a down-counter. The grammar designed for the N-bit Up–Down Counter is shown in Listing 5.

**Table 2:**
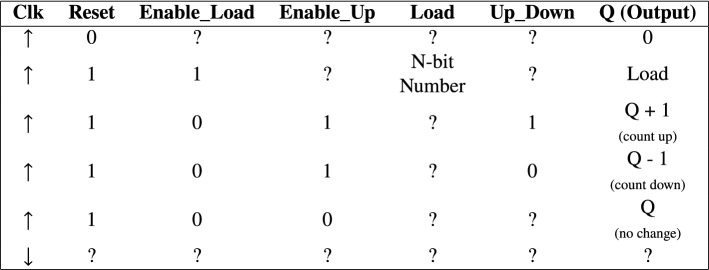
N-bit Up–Down counter transition table

The symbols $\uparrow$ and $\downarrow$ represent positive and negative transitions of the clock respectively. The ? symbol represents don’t care bits

### Testing

The selected default bit-width sizes of the parameterized circuits are high, rendering exhaustive testing infeasible. Furthermore, lexicase selection, our preferred selection method for digital circuit evolution, becomes computationally expensive with increasing number of test cases. As a result, we sample our training and testing cases using two strategies as shown in  Table 4. First, we identify the corner test case(s) for each circuit problem, usually targeting the test vectors located at the boundaries of all the possible test vectors (truth table). Second, given that the corner cases have been identified, only a few samples of the remaining cases are required. For the combinational circuits, the total number of cases is 50. The number of the remaining training and testing cases were generated by uniformly random sampling (using $urandom() function in SystemVerilog) the internal test vectors within the input range of each circuit; thus, $$50 - { n\underline{o}}$$ of corner cases. The total number of cases for the N-bit Up–Down Counter is 26 (15 and 11 corner and remaining cases respectively). The remaining cases target the counting up and down operations (5 cases each); a single case to set the counter in an initial state by loading a pre-specified value prior to training and testing. The use of corner cases and sampled test cases ensures our testbenches have good coverage over all possible test vectors.

The total number of cases for the Selective Parity and Adder circuits is 100. The Selective Parity circuit can behave as an odd or even parity circuit based on the even_odd input signal. During training and testing, both behaviours are tested. The Adder’s carry_in input signal can be either a 0 or 1. In a similar approach, the Adder circuit is trained and tested with the same cases when the carry_in input signal is 0 and 1 (Table 3).

All obtained solutions are tested for functional accuracy. Given that the evolved solutions for the parameterized circuits cannot possibly be tested by instantiating them with every possible bit-width size, we choose input bit-width sizes less and greater than the input bit-width with which the circuits were evolved/trained with. Test case generation is done using the same procedure outlined in Table 4 for generating the training cases. For example, the Adder was tested using 32-bit + 32-bit and 256-bit + 256-bit test vectors as shown in  Table 4.

Experiments were ran on a Dell Precision 7520 (laptop) configured with a RAM size of 64 GB and an Intel Xeon CPU (E3-1545M v5) with 4 CPU cores and a base frequency of 2.90 GHz.

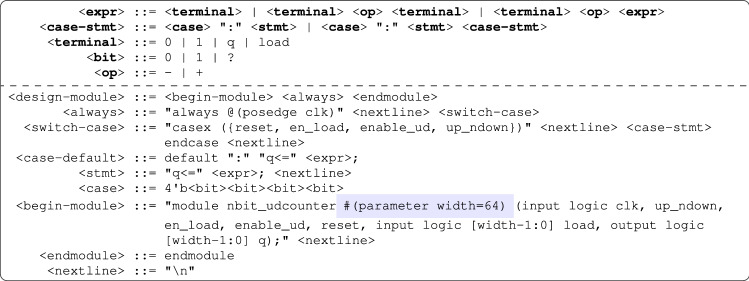



**Table 3:**
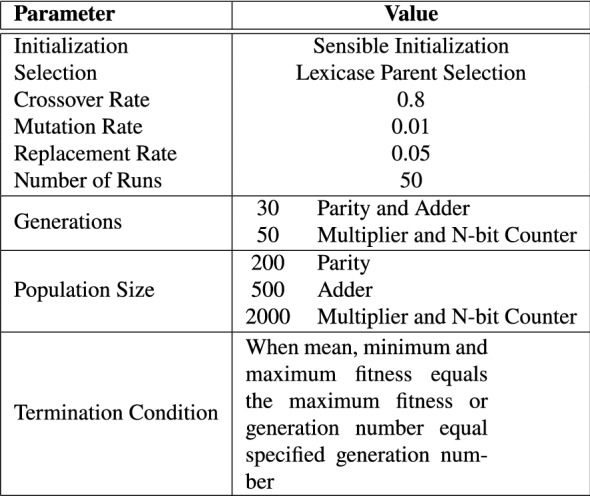
Evolutionary run parameters

In bold are the default population sizes used for the actual experiments

### Evolutionary Parameters

Preliminary experiments were conducted to determine reasonable parameter values for the population size and number of generation. Based on the preliminary results obtained, 30 generations and a population size of 500 are used for the parity and adder circuits; 50 generations and 2000 population size are used for the multiplier and Up–Down Counter circuits. All other parameters remain the same across all benchmark problems. Table 3 details the operators and parameters used in the experimental setup.

**Table 4:**
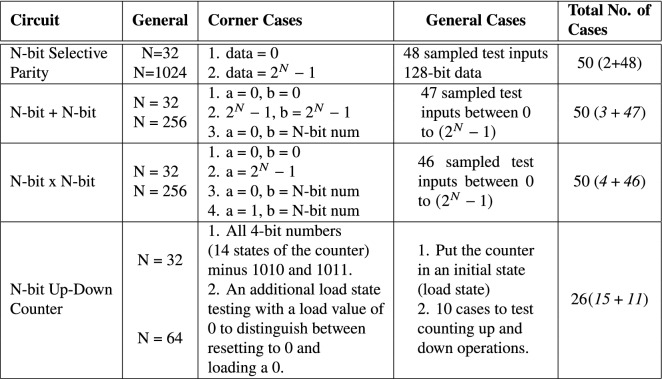
Training cases for benchmark problems

*a* and *b* refers to addends for the adder circuit. For the multiplier circuit, *a* and *b* are multiplier and multplicand respectively. *N* = bit-width

## Results and Discussion

Results obtained from our experiments are shown in this section. Additionally, we compare our results to those obtained by state-of-the-art approaches. In summary, based on results obtained, we conclude that the use of GE and an HDL (SystemVerilog) is capable of evolving complex combinational and sequential circuits, which answers the research questions set out in Sect. 1. However, it should be noted that when comparing with state-of-the-art approaches, despite our evolved circuits requiring less gates to realize in silicon, the optimization of circuits evolved at an abstract level like RTL is largely dependent on the robustness of the synthesis tool.

The rest of the section discusses the evolutionary performance of each benchmark circuit, success rate, analyzes of the effect of input sizes on circuit evaluation and compares of our results to state-of-the-art methods.

### Evolutionary Performance of Each Benchmark Circuit

**Fig. 2:**
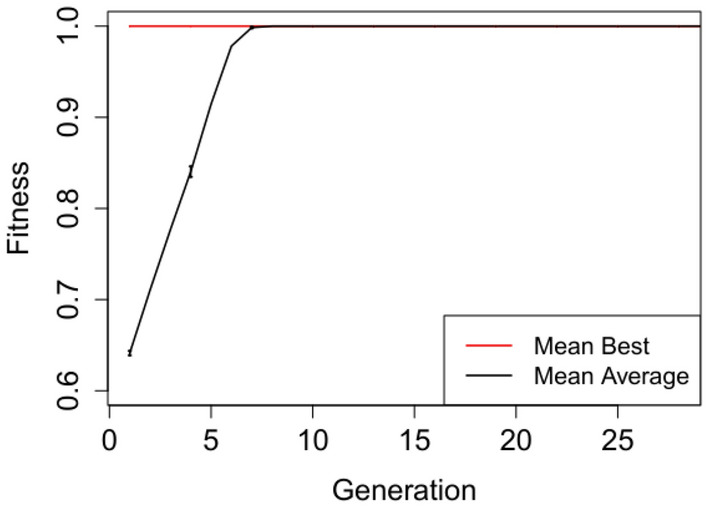
Mean best and mean average with error bars for N-bit Parity Generator Grammar A

**Fig. 3:**
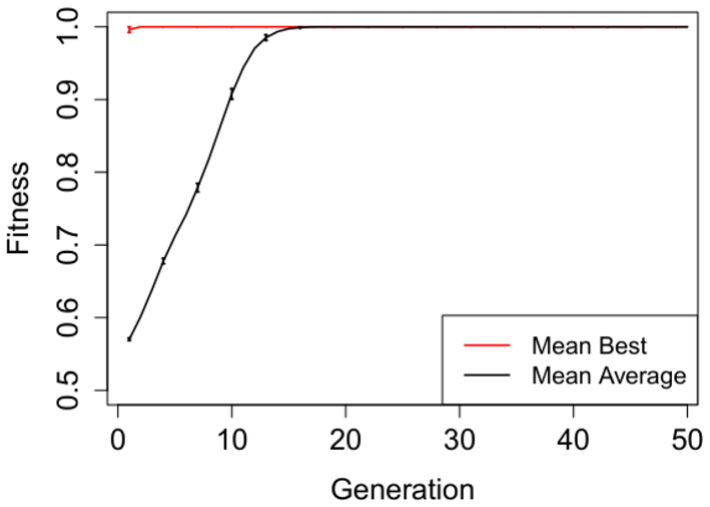
Mean best and mean average with error bars for N-bit Parity Generator Grammar B

**Fig. 4:**
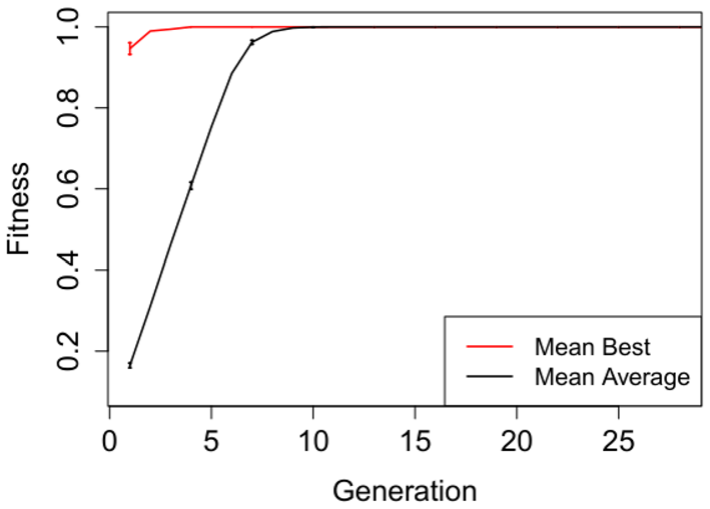
Mean best and mean average with error bars for N-bit + N-bit Adder

**Fig. 5:**
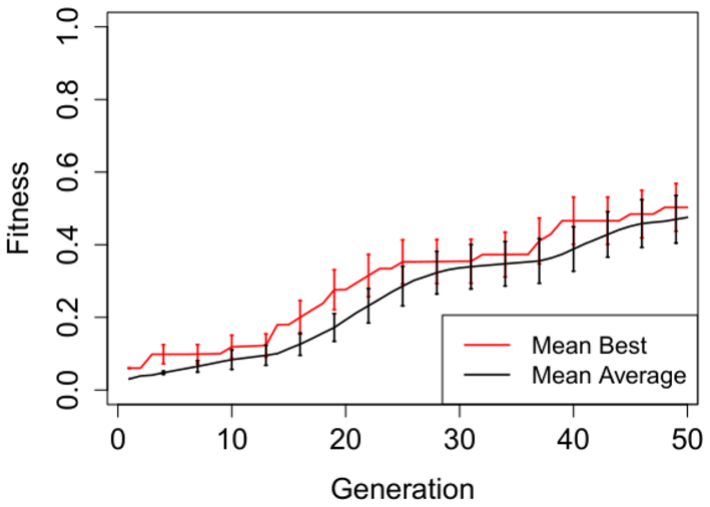
Mean best and mean average with error bars for N-bit $\times$ N-bit Multiplier

**Fig. 6:**
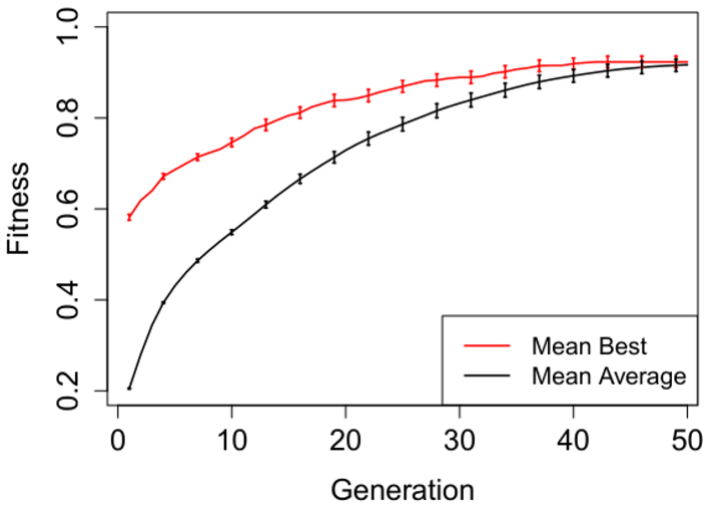
Mean best and mean average with error bars for N-bit Up-Down Counter

In Figures 2, 3, 4, 5 and 6 we plot the mean best and mean average fitnesses across the 50 independent runs conducted for each benchmark problem, in order to visualize evolutionary performance across generations.

Figures 2 and 3 show the plot for the Selective Parity Grammar A and Grammar B respectively. Both grammars attained maximum mean fitnesses from the initial generation, revealing the triviality in evolving parity generator circuits at this level of abstraction. Fully functional Selective Parity circuits were created via initialization without resorting to any form of evolution. The performance difference between the two grammars from these plots is that the Selective Parity Grammar B obtained lower mean average fitnesses than the Selective Parity Grammar A.

Evolution attains maximum mean best fitness within the first five generations for the adder circuit as observed in Figure 4. The starting mean average fitness is approximately 18% of the maximum fitness and increases rapidly to attain maximum mean fitness from generation 9 onwards. Both the mean best and mean average fitnesses for the Multiplier and N-bit Up–Down Counter progresses steadily, but do not attain maximum mean fitnesses. Furthermore, there exist short to non-existent error bars for the Selective Parity and Adder circuits in Figs. 2,  3 and 4 respectively, indicating little variability. The Up–Down Counter in Fig. 6 has non-existent to short error bars as the evolution progressed. For the multiplier circuit in Fig. 5, short error bars are observed in the earlier generations and widens gradually in subsequent generations, signifying increasing variability in individual fitness values compared to the mean fitness value.

### Success Rate

Fifty independent runs were conducted for each benchmark problem. The success rate for each problem is shown in Table 5. We define a successful run as an independent evolutionary run that evolves an optimal solution.

The N-bit Selective Parity (both Grammar A and B) and N-bit $+$ N-bit Adder circuits obtained a 100% successful runs. The N-bit $\times$ N-bit Multiplier and N-bit Up–Down Counter attained 46% and 48% successful runs respectively. Based on the population size and success rate of each circuit problem, the N-bit Selective Parity benchmark is the least difficult problem followed by the N-bit $+$ N-bit Adder. The N-bit $\times$ N-bit Multiplier and N-bit Up-Down Counter were the most challenging to evolve, requiring a higher population size.

**Table 5:**
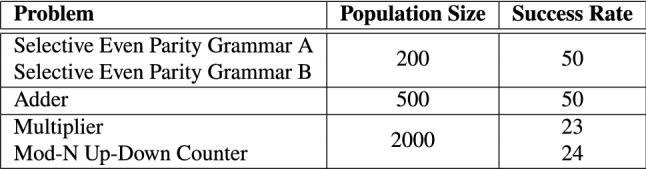
Success rate (Out of 50 Independent Runs)

### Effect of Input Sizes on Experiment Duration

Equally of importance is the effect of increasing input sizes (bit-width) on evaluation time. In order to make this assessment, we conducted separate experiments using smaller input-width sizes: *N* = 32 for Adder, Multiplier and Up–Down Counter circuits; *N* = 64 for the Selective parity circuit as shown in Table 6. A Wilcoxon test was then performed on the simulation time.

Increasing bit-width sizes of Selective Parity Grammar A, N-bit $+$ N-bit Adder and N-bit $\times$ N-bit Multiplier circuits has a significant effect on the evaluation time of circuits. In the case of Selective Parity Grammar A, recall we used bitwise operators together with a generate for-loop, as a result it requires a minimum of width evaluation events each time an input changes to generate the parity. However, Selective Parity Grammar B uses reduction operators that take a vector (multi-bit input), apply the respective operation to each individual bit and return a single bit as output. Reduction operators execute in a single evaluation event. Therefore, the use of reduction operators takes significantly less time to simulate Selective Parity circuits created with Grammar B. The Adder performs more additions as the input bit-width sizes increases. Similarly, the multiplier performs several additions to sum up the partial products. As a consequence, the significant increase in evaluation time as the input bit-width size increases for these circuits. In the case of the N-bit Up–Down Counter, no significant difference is observed because we did not exhaustively test the counting up and down operations over the entire input range. However, we do observe a slight difference in the mean experiment duration, signifying that the increase in input bit-width size does have some effect on the experiment duration.

Based on these observations, we suggest the choice of operators to be incorporated into grammars be carefully selected. Furthermore, selecting a reasonable default input bit-width size when evolving parameterized circuits can potentially reduce the experimental time. For example, an input bit-width size of 10 is reasonable for evolving a parameterized adder compared to a bit-width size of 1. A 1-bit + 1-bit Adder has only four test cases which may not be sufficient to properly train a parameterized Adder.

**Table 6:**
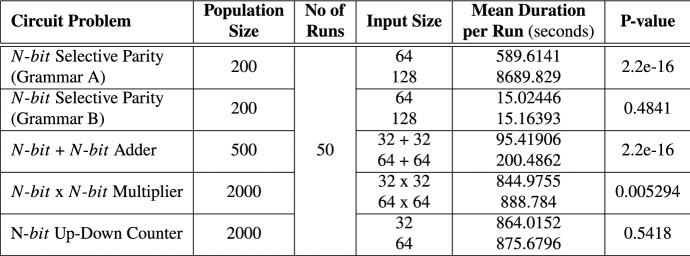
Effect of input size on experiment duration

**Table 7:**
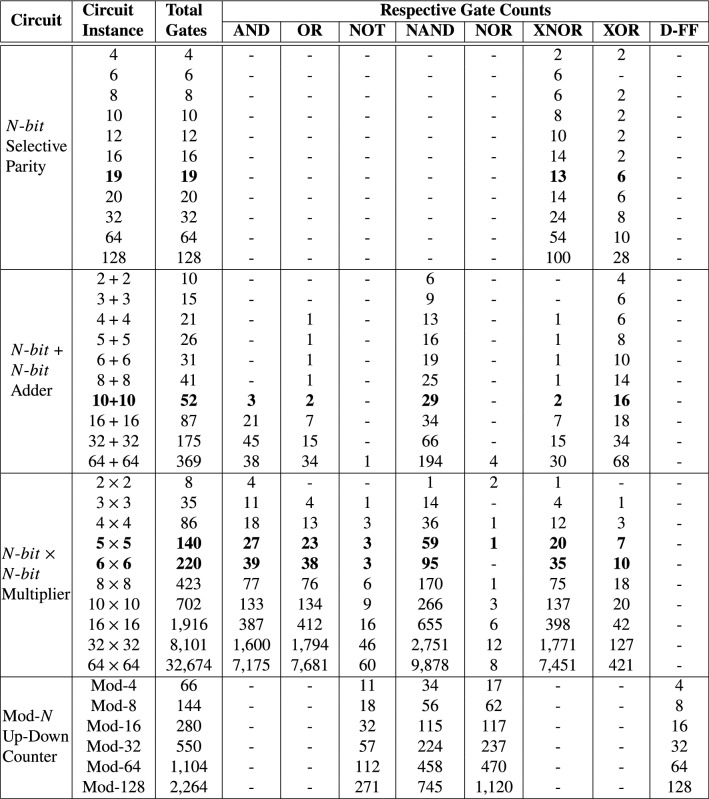
Number of logic gates required to realize a representative solution of each benchmark circuit evolved

**Table 8:**
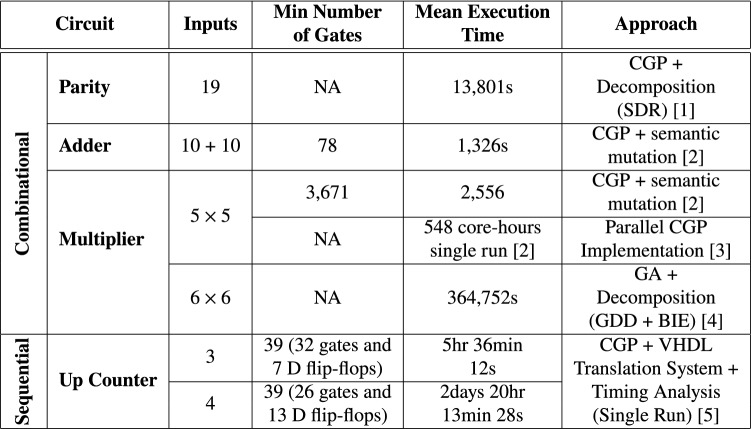
Most complex fully functional parity, adder, multiplier and counter circuits evolved

D-FF represent D flip-flop. In bold are the circuit instances used for comparing the gate count of our evolved circuits to those evolved by state-of-the-art approaches in Table 8

### Comparison with Literature

In Table 7, we synthesized a representative solution per benchmark problem to obtain the respective number of gates required to realize each circuit functionality in silicon. Given that all evolved circuits are parameterized, different circuit instances with different bit-width sizes were created for each and synthesized as shown in Table 7. The synthesis tool of choice is Yosys [1]. Yosys is an open source framework for Verilog RTL synthesis. Note that synthesis using commercial tools such as Quartus Prime, Vivado etc. may provide different gate counts. We compare our results (gate counts in Table 7 and mean execution time in Table 6) to the most complex circuits evolved in literature captured in Table 8.

Comparing the number of gates required to realize the respective circuits evolved by state-of-the-art approaches in Table 8 to our evolved circuits in Table 7, GE-evolved circuits require fewer gates. Similarly, less simulation time is required to evolve circuits (in Table 6) compared to state-of-art approaches in Table 8.

Since no N-bit Up-Down Counter has been evolved based on our literature review, in order to make a comparison to the 3-bit and 4-bit Up Counters in Table 8, we modified our evolved Up-Down Counter to disable the count down operation before synthesis. Our synthesized 3-bit requires 38 gates and 3 D-FFs; the 4-bit Up Counters require 46 gates and 4 D-FFs. Comparing these gate and D-FF counts to the number of gates and D-FFs to the Up Counter in Table 8, our resultant Up Counters use less number of D-FFs but require more gates. The number of D-FFs are consistent with the rule of thumb that an N-bit Counter requires an N number of flip-flops. However, our Up Counter comparison isn’t entirely fair as no specification on the number of inputs or transition table is provided for the evolved 3-bit and 4-bit Up Counters in [26], since they affect gate count.

## Conclusion

In this work, we have shown that designing circuits at a higher level of abstraction, *RTL*, than gate level, allows for the design of significantly larger circuits than existing approaches in literature. Fully functional N-bit selective parity, N-bit + N-bit adder, N-bit $\times$ N-bit multiplier and N-bit Up-Down counter circuits have been successful evolved, representing the most complex circuits evolved in literature to date. The availability of expressive programming constructs and operators in SystemVerilog aided greatly in the evolution of these circuits. Furthermore, our approach recorded better mean execution time and requires less number of gates after synthesis compared to state-of-the-art results.

The evolved circuits are parameterized, meaning once a circuit is evolved, different circuit instances of varying input sizes can be obtained, simply by specifying the desired input sizes without requiring a re-run of experiments compared to existing methodologies. The work also established how appropriate choice of operators can significantly reduce simulation time in the case of the selective parity benchmark. Also, using reasonable input bit-width sizes to evolve parameterized circuits generally reduces the evaluation time, while the evolutionary performance remains the same.

Despite the availability of high level programming constructs in HDLs which helps to evolve complex circuits, we acknowledge certain circuit problems require decomposition. Decomposition is a normal practice in industry. HDLs support hierarchical modelling for the design of large and complex circuits. In hierarchical modelling, design modules in lower layers are included in design modules in higher layers. Communication between circuits are established through the input and output ports of the circuits. Very large complex circuit will be slow to test. Therefore, the conventional generate and test approach of standard evolutionary systems may not suffice. A more efficient approach that will exploit the power of modern processors will be of high priority when we begin to venture into industrial scale circuit designs.

In a typical circuit design flow, SystemVerilog evolved circuits are advanced to the synthesis phase, where synthesis tools are used to obtain the equivalent gate-level representation, as we did using Yosys. As a result, circuit optimization is dependent on the robustness of open-source or commercial synthesis tools. However, progress made in Evolutionary Optimization of Digital Circuits, a sub-field of EHW which focuses on the optimization of functional circuits, have obtained competitive results compared to conventional synthesis tools [52]. Most of the evolutionary optimization of circuits use CGP. Therefore, a merger of GE and CGP for functional evolution and circuit optimization respectively will be the starting point of realizing evolutionary circuit design tool chains.
